# Dynamic Evolution Analysis of Desertification Images Based on BP Neural Network

**DOI:** 10.1155/2022/5645535

**Published:** 2022-03-17

**Authors:** Guanyao Lu, Dan Xu, Yue Meng

**Affiliations:** School of Environmental and Chemical Engineering, Foshan University, Foshan 528000, Guangdong, China

## Abstract

In recent years, with the rise of artificial intelligence, deep neural network models have been used in various image recognition researches. Land desertification is a major environmental problem facing the world at present, and how to do a good job in dynamic monitoring is particularly important. For remote sensing images, this paper constructs a GA-PSO-BP analysis model based on BP neural network, genetic algorithm, and particle swarm algorithm and compares the classification training accuracies of the four models of BP, GA-BP, PSO-BP, and GA-PSO-BP; GA-PSO-BP was selected for dynamic analysis of desertification images, and the results showed the following: (1) By comparing the regional classification training accuracies of the four models of BP, GA-BP, PSO-BP, and GA-PSO-BP, the GA-PSO-BP neural network remote sensing image classification method proposed in this paper is simple and easy to operate. Compared with traditional remote sensing image classification methods and traditional neural network classification methods, the classification accuracy of remote sensing effects is improved. (2) Carrying out desertification analysis on remote sensing images of Horqin area, from 2010 to 2015, the desertified land area in the test area increased by 1.56 km^2^; from 2015 to 2020, the desertified land area in the test area decreased by 1.131 km^2^, and the desertified land in the test area from 2010 to 2020 showed a trend of increasing first and then decreasing, which is consistent with the actual situation. The GA-PSO-BP remote sensing image classification model has a good performance portability.

## 1. Introduction

In the early 1970s, the concept of desertification was put forward. Land desertification is also called land degradation, which is caused by the fragility of the natural environment and unreasonable human production activities. Natural factors for the rapid spread of desertified land include drought and lack of water, uncontrolled grazing destroying natural vegetation, wind erosion, and water erosion; human factors include excessive logging and waste of water resources. Once the land shows signs of desertification, the most obvious manifestation is the gradual decline or even loss of soil productivity. The phenomenon of land desertification occurs on a global scale, affecting about 25% of the world's land [1].

The prerequisite for desertification land management is real-time and accurate judgment and quantitative analysis of desertification land changes. The two most important links are land classification and land change monitoring. Using remote sensing data and remote sensing technology to classify land is currently the main land use classification method [2]. The two methods of BP neural network and convolutional neural network also belong to the category of remote sensing supervision classification [3]. Among them, the experimental effect of BP neural network in the classification of standard remote sensing images and high score images [4], compared with traditional methods, is better in terms of accuracy. Han et al. [5] and Anwer et al. all borrowed the idea of BP neural network to classify the heterogeneous images in the study area; Han et al. built a new classification based on the classification results of the BP neural network. The adaptive classifier improves the classification accuracy; Anwer et al. [6] used the BP neural network of RBF to classify the TM images in the study area. In the research process, Park et al. [7] tried to use two different data source processing methods, prefusion and postfusion, to explore whether the data source has an impact on CNN classification. At the same time, in terms of change monitoring, remote sensing technology has many advantages. Remote sensing images contain a large amount of information, a wider range of investigations, and a faster survey work. Through the comparative research of multitemporal information, dynamic monitoring and trend prediction can be realized. Tasgetiren et al. [8] used differential DEM algorithm to monitor the topographic changes and topographic changes of the study area. Haque and Basak [9] combined the change vector analysis (CVA) method with the postclassification method and applied it to 4 datasets, and the accuracy was higher than the change monitoring accuracy of the algebraic method alone. Lal et al. [10] proposed a multi-time-domain remote sensing image change monitoring method based on sparse fusion and constrained *k*-means clustering was proposed. It adopts the idea of early fusion and later classification and compares the fusion monitoring results after classification, and the accuracy is improved.

This paper uses multispectral remote sensing images as the experimental data source, uses different classification methods, and introduces genetic algorithm (GA) and particle swarm optimization (PSO) algorithm to classify images in the study area and test area. The experimental results are compared with each other and the structure selected is simpler and the simulation error is small. The classification method is used to classify the images in the test area. Finally, the difference method is used to monitor the changes of the classified images, so as to achieve the purpose of macrocontrolling the overall change trend of the desertified land [11]. The method proposed in this paper can effectively make up for the shortcomings of traditional change monitoring methods and provide a new method and idea for desertification land change monitoring.

## 2. Related Work

### 2.1. BP Neural Network

**Figure 1:**
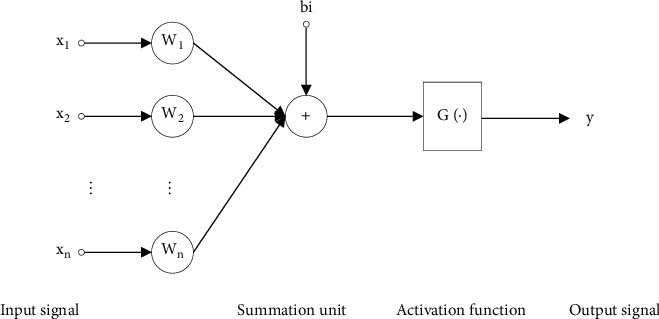
Structure diagram of a single neuron.

BP neural network is a self-adaptive nonlinear dynamic network system composed of neuron models, thresholds, activation functions, and so forth connected to each other [12]. The structure of a single neuron model is shown in Figure 1.

**Figure 2:**
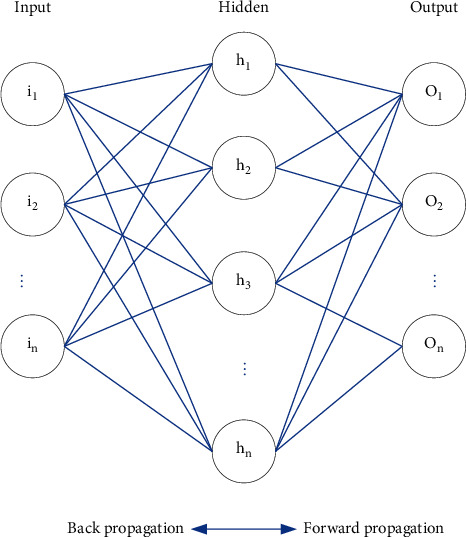
Neural network structure diagram.

Among them, the weight of the input signal is represented by *W*, the bias is represented by *b*, and *G*(·) represents the activation function. The common feedforward neural network structure is divided into single layer and double layer, as well as recursive formula [13]. The basic structure of a multilayer feedforward neural network is from front to back: input layer (one layer) ⟶ hidden layer (1 or more layers) ⟶ output layer (one layer). BP neural network belongs to the category of multilayer feedforward network. The input signal conforms to the principle of forward propagation, and the error generated in the training process conforms to the principle of backpropagation. It is widely used in the field of remote sensing image processing. The training error backpropagation, in short, takes the specified training error as the target, and the training error that does not meet the target is continuously adjusted and updated from the output layer to the input layer, until the target error is reached and the iterative update is stopped. The complete structure of the BP neural network model is shown in Figure 2. Two parts are described in detail below: signal forward propagation and error backward propagation.

Among them are the input signals (*i*_1_ and *i*_2_), the connection threshold between the first layer and the second layer (*b*_1_ and *b*_n_), the neurons in the hidden layer (*h*_1_ and *h*_2_), and the neurons in the output layer (*o*_1_ and *o*_2_). When determining the parameters of the BP neural network, you need to set the learning rate. The setting of the learning rate has only a reference range. If the value is too large, the network will oscillate, which may lead to failure to converge. If the value is too small, it will also greatly increase the time required for network convergence. At the same time, the BP neural network cannot memorize all the weight thresholds used in the learning process. Each iteration will overwrite the previous record. When there are new input samples, the weight threshold matrix of BP neural network will change accordingly, disrupting the trained sample features and classification rules, interfering with network training, and reducing the efficiency of learning.

### 2.2. Genetic Algorithm

**Figure 3:**
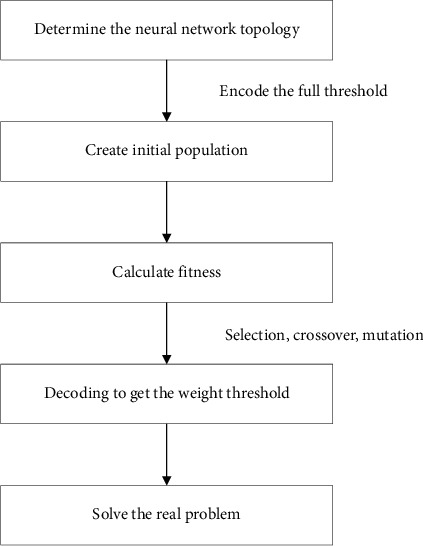
Flow chart of the genetic algorithm.

Genetic algorithm is an optimization algorithm that simulates biological evolution, that is, chromosome duplication and mutation in organisms [14]. The main purpose is to select and globally optimize the objective function according to the “survival of the fittest” principle. The chromosome is the basic component, representing the vector of the objective function [15]. Compared with traditional optimization methods (enumeration, heuristic, etc.), genetic algorithm is based on biological evolution and has good convergence, less calculation time, and high robustness when calculation accuracy is required.  Figure 3 shows the flow chart of the genetic algorithm.

1. Coding: Use a suitable coding method for vector coding, and this article uses binary coding.
2. Create the initial chromosome community: Use the creation function and give the community size (*N*) to generate the initial community.
3. Calculate individual fitness: Determine a reasonable fitness function, and calculate the fitness value of each chromosome in the community *N* according to the fitness function. The fitness value is sorted from large to small and divided into three parts.
4. Selection: According to the calculation result of step 3, the part of the chromosome with higher fitness will be kept after copying.
5. Crossover: According to the calculation result of step 3, some chromosomes with moderate fitness are retained after crossover.
6. Mutation: According to the calculation result of step 3, some chromosomes with lower fitness will be retained after mutation.

The above steps 1–6 need to be iterated continuously, which is in line with the principles of biological genetic evolution. The purpose is to preserve the excellent genes in the population and inherit them to the offspring, so that the offspring have a higher average fitness and individual fitness. The genetic algorithm is simple, universal, and robust [16], which can search from different directions at the same time, which improves the global search ability to a certain extent.

### 2.3. Particle Swarm Algorithm

Particle swarm algorithm is also one of the commonly used global search algorithms [17]. The algorithm is proposed after studying and analyzing the predation behavior of birds [18]. The basic idea is to designate a certain point in the target area as the target food, and a flock of birds randomly search for the target food in the target area. Birds search without any prior knowledge about the target food, and it is necessary to quickly determine the search range of the bird group closest to the target food.

**Figure 4:**
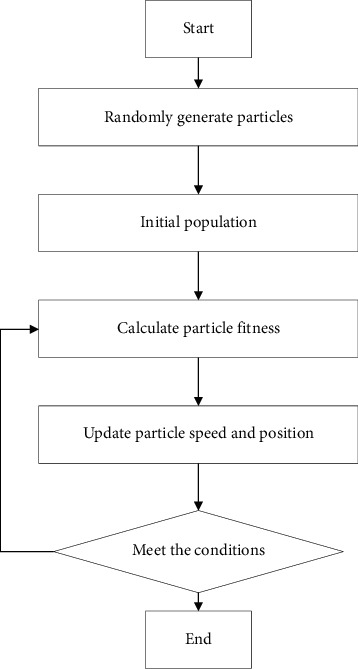
Particle swarm algorithm flow chart.

Compared with other optimization algorithms, the particle swarm optimization algorithm has a simple structure and can effectively reduce the time required for network convergence [19]. At present, it is widely used in many fields such as pattern recognition, image processing, and function optimization [20].  Figure 4 shows the flow chart of particle swarm algorithm.

1. Randomly generate particle swarms: The size of the particle swarm and the position and velocity of the particles need to be set in advance, as well as other parameters.
2. Calculate the fitness value: Calculate the fitness value *F* of the particle based on the prescribed fitness function; compare *F* with the individual extreme value; if *F* > the individual extreme value, then replace the current individual extreme value with *F*. Globally the update of the extreme value is the same.
3. Update particle speed and position: Update the particles according to the two following update formulas.
4. Iterative judgment: If the current individual extreme value and the global extreme value meet the end conditions, it will automatically exit the iteration and complete the optimization calculation process. Otherwise, continue to iterate until the number of iterations reaches the maximum set before initialization.

The velocity update formula of a single particle in the particle swarm algorithm is as shown in formula (1):

(1) $$\begin{array}{c} {\text{new}}_{-}V_{i}=\omega \cdot v_{i}+c1\cdot \text{rand}\left(\right)\left(\;\right)\cdot \left(p\text{best}-P_{i}\right)+c2\left(\;\right)\cdot \text{rand}\left(\right)\cdot \left(g\text{best}-P_{i}\right), \end{array}$$

where *ω* is the inertia factor, *v*_*i*_ is the current velocity of the particle, and *P*_*i*_ is the current position of the particle. The location update formula is as follows:

(2) $$\begin{array}{c} {\text{new}}_{-}P_{i}=P_{i}+{\text{new}}_{-}v\cdot t. \end{array}$$

In the above equation, *t* is generally set to 1 by default. The PSO algorithm needs fewer parameters to adjust, and the particles have memory capabilities [21], and at the same time it can reduce the time required for network convergence.

## 3. GA-PSO-BP Model

This paper proposes a remote sensing image classification method and change monitoring based on a BP neural network model optimized by GA and PSO. The purpose is to effectively alleviate the instability of BP neural network memory learning, reduce the time required for network convergence, and ensure the classification accuracy of remote sensing images.

**Figure 5:**
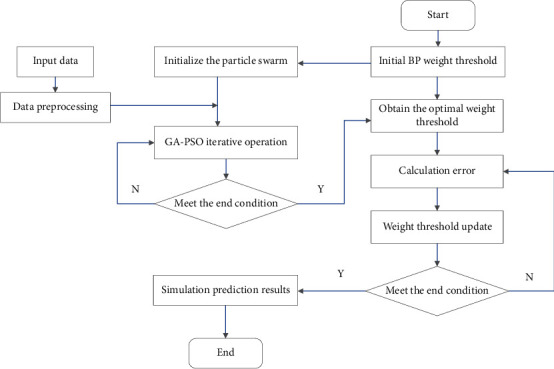
GA-PSO-BP neural network process.

The genetic algorithm is used to further optimize the particles of the particle swarm algorithm. The optimization content includes particle speed and position, and the optimization operation includes copy, crossover, and mutation, which increases the complexity of the particle swarm algorithm and ensures that the particle swarm algorithm is executed. In the process, it will not fall into a local minimum, which ensures that the particles can complete the network optimization. Make full use of the memory characteristics of the particle swarm algorithm to ensure that the optimal solution is memorized every time in the search process. The GA-PSO-BP process is shown in Figure 5, and its specific implementation steps are as follows:

1. Initialization. Need to specify the number of particles of the randomly initialized population A. The remaining parameters that need to be initialized include various parameters used in the process of GA and PSO operations, as well as the maximum number of iterations *N* of the algorithm.
2. Using the particle swarm algorithm, first perform optimization on population A, and the criterion for judging whether it needs to be updated is the fitness function, and the content of the update is the particle speed and position.
3. The principle of particle sorting: the fitness values are arranged in order from large to small, and they are divided into three parts. Population A1 represents the part of the particles with the best fitness, population A2 represents the part of the particles with the fitness in the middle, and population A3 represents the part of the particles with the smaller fitness.
4. A1 (copy), A2 (crossover), and A3 (mutation).
5. Update gbest and ybest.
6. Repeat the above steps 2–5 until the number of iterations reaches the set maximum number or the objective function reaches the convergence accuracy.

Among them, in the GA-PSO iteration process, multiple parameters need to select appropriate calculation update formulas. The following is an introduction to the selection of important ones.

For fitness function, the absolute value of the error between the predicted value of the BP neural network and the set value is used to construct the fitness function. The calculation formula is

(3) $$\begin{array}{c} f=\sum_{i=1}^{n}\left|y_{i}-o_{i}\right|, \end{array}$$

where *n* is the total number of output nodes; *y*_*i*_ is the set value of the *i*-th node; *o*_*i*_ is the predicted value of the *i*-th node. For selection operator, the roulette method is used. For individual *i* in the particle swarm size *N*, the selection probability *P*_*s*_ is

(4) $$\begin{array}{c} P_{s}=\frac{\left(1/f\right)}{\left(\sum_{i=1}^{N}1/f\right)}. \end{array}$$

For crossover operator, equations (5) and (6) are used to perform crossover operations on the positions and velocities of particles *i* and *j*.

(5) $$\begin{array}{c} \left\{\begin{array}{c} X_{i\;d}^{k+1}=\lambda_{1}X_{i\;d}^{k}+\left(1-\lambda_{1}\right)X_{j\;d}^{k},\\ X_{j\;d}^{k+1}=\lambda_{1}X_{j\;d}^{k}+\left(1-\lambda_{1}\right)X_{i\;d}^{k}, \end{array}\right. \end{array}$$

(6) $$\begin{array}{c} \left\{\begin{array}{c} V_{i\;d}^{k+1}=\lambda_{2}V_{i\;d}^{k}+\left(1-\lambda_{2}\right)V_{j\;d}^{k},\\ V_{j\;d}^{k+1}=\lambda_{2}V_{j\;d}^{k}+\left(1-\lambda_{2}\right)V_{i\;d}^{k}. \end{array}\right. \end{array}$$

In formulas (5) and (6), *λ*_1_,  *λ*_2_ is a random number in [0, 1]. For mutation operator, equations (7) and (8) are used to perform mutation operations on the position and velocity of particles *i* and *j*.

(7) $$\begin{array}{c} X_{i\;d}^{k+1}=\left\{\begin{array}{c} X_{i\;d}^{k}+\left(X_{i\;d}^{k}-X_{\text{max}}\right)f\left(g\right),\;\alpha_{1}\geq 0.5,\\ X_{i\;d}^{k}+\left(X_{\text{min}}-X_{i\;d}^{k}\right)f\left(g\right),\;\alpha_{1}<0.5, \end{array}\right. \end{array}$$

(8) $$\begin{array}{c} V_{i\;d}^{k+1}=\left\{\begin{array}{c} V_{i\;d}^{k}+\left(V_{i\;d}^{k}-V_{\text{max}}\right)f\left(g\right),\;\alpha_{2}\geq 0.5,\\ X_{i\;d}^{k}+\left(V_{\text{min}}-V_{i\;d}^{k}\right)f\left(g\right),\;\alpha_{2}<0.5, \end{array}\right. \end{array}$$

(9) $$\begin{array}{c} f\left(g\right)=\alpha_{3}\left(1-\frac{g}{G}\right), \end{array}$$

where *X*_max_, *X*_min_ are the upper and lower bounds of the particle position; *V*_max_, *V*_min_ are the upper and lower bounds of the velocity; *α*_1_, *α*_2_, *α*_3_, is a random number, usually between [0, 1]; *g* represents the current evolution times; *G* is the largest algebra that can evolve. Inertia weight *ω* adopts adaptive *ω* adjustment method, as shown in the following equation:

(10) $$\begin{array}{c} \omega =\omega_{\text{max}}-k\left[\frac{\left(\omega_{\text{max}}-\omega_{\text{min}}\right)}{k}\right], \end{array}$$

where *ω*_max_, *ω*_min_ are the upper and lower bounds of the inertia weight, *k* is the number of current iterations, and *K* is the total number of iterations.

## 4. Dynamic Analysis of the Impact of Desertification

### 4.1. Operating Environment

The experiment is based on MATLAB 2018a development platform and MATLAB 2018a neural network toolbox. All are constructed by *M* language. The toolbox contains a large number of functions closely related to practical applications. When the corresponding problem needs to be solved, it can be called directly without rewriting (Table 1).

### 4.2. Sample Data Processing

Normalizing the input data can effectively avoid the redundancy of the data involved in the calculation. The expected output of the network corresponds to the true label of each type of feature. Before the training starts, the training samples must first be transformed into a one-dimensional vector *I*.

(11) $$\begin{array}{c} I_{kl}=\frac{I_{kl}-I_{\text{min}}}{I_{\text{max}}-I_{\text{min}}}. \end{array}$$

In the above equation, *I*_*kl*_ represents the pixel value of the *l*-th image feature in the *k*-th sample, and the maximum and minimum values within the range of the *l*-th image feature pixel value are represented by *I*_max_ and *I*_min_. Based on visual interpretation and judgment of image data combined with field investigation, this paper divides the ground objects in the training area into 6 categories: water, buildings, shadows, bare land, roads, and cultivated land. Suppose that the expected output vector of the BP neural network is *O*:

(12) $$\begin{array}{c} O=\left[\begin{array}{cc} \begin{array}{ccc} 1 & 0 & 0\\ 0 & 1 & 0\\ 0 & 0 & 1 \end{array} & \begin{array}{ccc} 0 & 0 & 0\\ 0 & 0 & 0\\ 0 & 0 & 0 \end{array}\\ \begin{array}{ccc} 0 & 0 & 0\\ 0 & 0 & 0\\ 0 & 0 & 0 \end{array} & \begin{array}{ccc} 1 & 0 & 0\\ 0 & 1 & 0\\ 0 & 0 & 1 \end{array} \end{array}\right]. \end{array}$$

Each column of the matrix represents a category, where the expected output of the water body is ${\left[\begin{array}{cc} \begin{array}{ccc} 1 & 0 & 0 \end{array} & \begin{array}{ccc} 0 & 0 & 0 \end{array} \end{array}\right]}^{T}$, the expected output of the building is ${\left[\begin{array}{cc} \begin{array}{ccc} 0 & 1 & 0 \end{array} & \begin{array}{ccc} 0 & 0 & 0 \end{array} \end{array}\right]}^{T}$, and the expected output of the shadow is ${\left[\begin{array}{cc} \begin{array}{ccc} 0 & 0 & 1 \end{array} & \begin{array}{ccc} 0 & 0 & 0 \end{array} \end{array}\right]}^{T}$; the expected output of bare land is ${\left[\begin{array}{cc} \begin{array}{ccc} 0 & 0 & 0 \end{array} & \begin{array}{ccc} 1 & 0 & 0 \end{array} \end{array}\right]}^{T}$, the expected output of roads is ${\left[\begin{array}{cc} \begin{array}{ccc} 0 & 0 & 0 \end{array} & \begin{array}{ccc} 0 & 1 & 0 \end{array} \end{array}\right]}^{T}$, and the expected output of cultivated land is ${\left[\begin{array}{cc} \begin{array}{ccc} 0 & 0 & 0 \end{array} & \begin{array}{ccc} 0 & 0 & 1 \end{array} \end{array}\right]}^{T}$.

### 4.3. Model Parameter Setting

#### 4.3.1. BP Neural Network

*(1) Selection of Input and Output*. The setting of the number of hidden layers and the number of nodes is the key to the network and has an impact on the learning and generalization capabilities of the network. The number of hidden layers needs to be determined through experiments or empirical rules and the principle cannot be set blindly, which may cause the network to be too computationally intensive. This may cause the network to be too computationally expensive, because each piece of sample data will have unavoidable noise, and the neural network will be affected by noise to varying degrees while learning the characteristics of the sample data. So, under normal circumstances, you can adjust the number of hidden layer nodes instead of increasing the number of hidden layers.

When using BP neural network to classify remote sensing images, the number of bands of remote sensing data is the number of nodes in the input layer. The sample data in this article has only 3 multispectral bands; as long as it contains enough spectral feature information of the ground features, the number of nodes in the input layer is determined to be 3. The number of nodes in the input layer is determined to be 3; the number of nodes in the output layer represents the number of land types specified in the experiment, and the number of nodes in the output layer is 6, and then the number of hidden layer nodes is determined according to the prior rules of the input layer and the hidden layer given by

(13) $$\begin{array}{c} n_{2}=2\times n_{1}+1. \end{array}$$

It is calculated that the number of nodes in the hidden layer of the experiment in this paper *n*_2_ is 7, and the network structure of the BP neural network is finally determined: 3-7-6 (input layer-hidden layer-output layer).

*(2) Selection of Activation Function*. The activation function adds nonlinear elements to the neural network. First, the activation function should satisfy the specified basic conditions. The activation function affects the time for the entire network to approach the target, and the prediction and generalization effect of the input samples. The sigmoid function is a common sigmoid function. After comparative experiments, the sigmoid function is finally determined as the network activation function in this paper.

*(3) Network Global Error*. Whether the global error can reach the target value is the basic criterion for judging the classification accuracy of the neural network. The setting of the global error should not only consider the actual situation of the network model but also consider the convergence speed of the network model. If the target setting is too small, the convergence speed will be reduced. It becomes very slow and cannot be used in practice; setting the target too large will lead to a substantial increase in the rate of misjudgment. Before setting the global error, specify 2-3 different global errors to conduct experiments. After the experiment, through comparison, determine the most suitable global error value. The global error TrainGoal selected in this paper is 0.000001.

**Figure 6:**
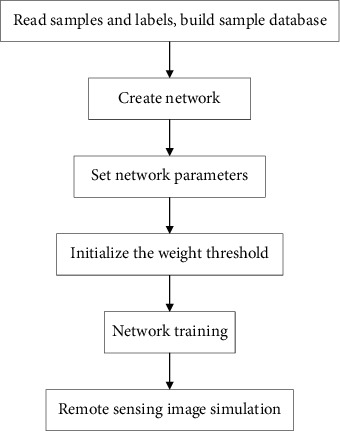
BP neural network remote sensing image classification process.

*(4) Learning Rate*. Determining a scientific and reasonable learning rate can ensure that there is no abnormal deviation from network errors. The setting range is [0.01 0.8]. If the learning rate is set too large, the network will lose stability during the training process. If the setting is too small, it will take too long for the network to reach the target value, which is more complicated for the large number of network layers. The circumstances and the requirements for learning rate are not the same. According to the adaptive gradient descent method, the learning rate in the training process can be controlled, and it can also be automatically adjusted according to the characteristics of the training situation. Finally, the adaptive gradient descent method is determined to effectively improve the performance and stability of the algorithm. Construct a BP neural network based on the above selected parameters, and perform remote sensing image classification experiments on the training area.  Figure 6 shows the BP neural network remote sensing image classification process.

#### 4.3.2. GA-BP Model

**Figure 7:**
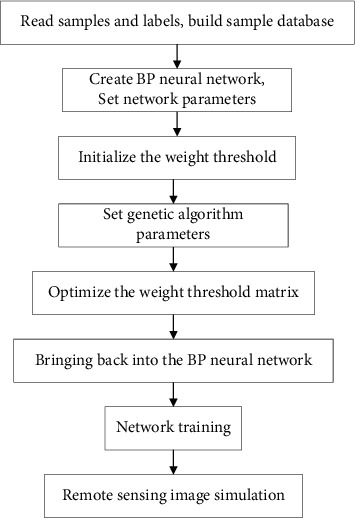
The remote sensing image classification process of the GA-BP optimization model.

Determining the basic parameters of BP neural network is the first task for GA optimization. This experiment still uses a BP neural network whose input layer-hidden layer-output layer is “3-7-6.” In addition, determine the three most important structural parameters: training function (traingda), adaptive gradient descent method, and global error (0.000001). After determining the structural parameters of the BP neural network, the main parameters of GA need to be determined, including population size (*M*), maximum number of iterations (*N*), crossover probability (*cp*), and mutation probability (*mp*). The population size of this experiment is set to *M* = 200, the maximum number of iterations *N* = 500, the crossover probability *cp* = 0.6, and the mutation probability *mp* = 0.06. According to the parameters selected previously, the GA optimized BP neural network is constructed, and the remote sensing image classification experiment is performed on the training area.  Figure 7 shows the GA optimized BP neural network remote sensing image classification process.

#### 4.3.3. PSO-BP Model

**Figure 8:**
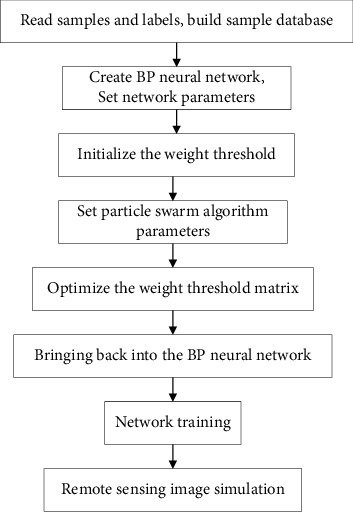
The remote sensing image classification process of the PSO-BP optimization model.

Determining the basic parameters of BP neural network is the primary task of PSO optimization. This experiment still uses a BP neural network whose input layer-hidden layer-output layer is “3-7-6.” In addition, determine the three most important structural parameters: training function (traingda), adaptive gradient descent method, and global error (0.000001). The most important parameters for the particle swarm algorithm are the particle swarm (*A*), the number of particles, the learning factor (*c*1 and *c*2), the inertia weight (*w*), and the maximum number of iterations (*N*2). In this experiment, the number of particles in particle swarm *A* is set to 200, the learning factors *c*1 and *c*2 = 2, the inertia weight *w* = 0.7, and the maximum number of iterations *N*2 = 500. Construct a PSO optimized BP neural network based on the above selected parameters, and perform remote sensing image classification experiments on the training area.  Figure 8 shows the PSO optimized BP neural network remote sensing image classification process.

#### 4.3.4. GA-PSO-BP Model

Determining the basic parameters of the BP neural network is the first step in the optimization of GA combined with PSO. This experiment still uses a neural network whose input layer-hidden layer-output layer is “3-7-6.” Determine the three most important structural parameters: training function traingda, adaptive gradient descent method, and global error (0.000001).

**Figure 9:**
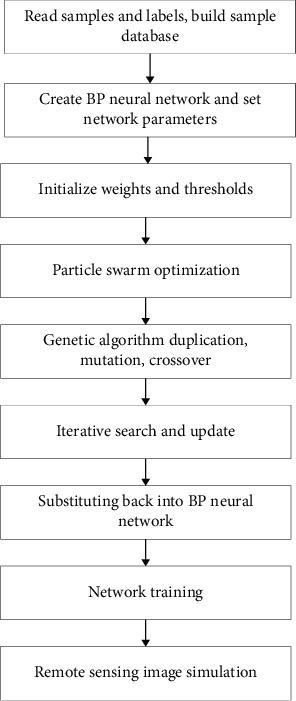
The remote sensing image classification process of the GA-PSO-BP optimization model.

For genetic algorithm, the population size of this experiment is *M* = 200, the maximum number of iterations is *N* = 500, the crossover probability *cp* = 0.6, and the mutation probability *mp* = 0.06. For the particle swarm algorithm, the number of particles in particle swarm *A* in this experiment is 200, the learning factors *c*1 and *c*2 = 2, inertia weight *w* = 0.7, and maximum number of iterations *N*2 = 500.  Figure 9 shows the GA-PSO-BP neural network remote sensing image classification process, which mainly includes network creation, parameter setting, preliminary optimization of particle swarm algorithm, block and combine genetic algorithm processing according to fitness value, generation optimization, updating individual extreme value and group extreme value, network training, and remote sensing image classification.

### 4.4. Evaluation of Model Training Results

Classify and train the four models of BP neural network and GA-BP, PSO-BP, and GA-PSO-BP. This paper uses the pixel-level confusion matrix evaluation method to calculate the Kappa coefficient and the overall accuracy (OA) to classify the quality of each model. For evaluation, the real number of pixels of the same land type within the specified area and the number of pixels with a certain confidence level and the number of pixels obtained by the statistics after classification by the classification algorithm are the basic elements to form a complete confusion matrix (formula (11)). In formula (11), the columns of the matrix represent the images after classification, and the rows of the matrix represent the number of pixel categories:

(14) $$\begin{array}{c} M=\left[\begin{array}{ccc} x_{11} & \cdots & x_{m1}\\ \vdots & \ddots & \vdots \\ x_{1m} & \cdots & x_{mm} \end{array}\right]. \end{array}$$

In the above equation, *m* is the total number of categories, and *x*_*ij*_ represents the number of pixels in the study area that should belong to category *i* but are classified as category *j*. The confusion matrix calculated by Arcgis is the basis for further analysis. The main evaluation indicators of this article are calculated based on the confusion matrix: OA and Kappa coefficient.

OA is calculated based on the total number of pixels that have been correctly classified, divided by the total number of pixels:

(15) $$\begin{array}{c} \text{OA}=\frac{\sum_{i=1}^{m}x_{ii}}{\sum_{i=1}^{m}\sum_{j=1}^{m}x_{ij}}. \end{array}$$

Kappa coefficient is calculated as

(16) $$\begin{array}{c} K=\frac{N\sum_{i=1}^{m}x_{ii}-\sum_{i-1}^{m}\left(x_{i+}x_{+i}\right)}{N^{2}-\sum_{i-1}^{m}\left(x_{i+}x_{+i}\right)}, \end{array}$$

where *N* represents the total number of pixels, *x*_*i*+_ represents matrix column sum, and *x*_+*i*_ represents matrix row sum.

The calculation result of the Kappa coefficient generally falls within the range of [−1, 1]. The specific description of the actual situation is that it needs to be distinguished by grouping the Kappa coefficients, so the Kappa coefficients are divided into 5 levels:

1. Falling in the range of [0.0, 0.20]: such a result is defined as keeping very low consistency with the actual result;
2. Falling within the range of [0.21, 0.40]: such a result is defined as maintaining general consistency with the actual result;
3. Falling within the range of [0.41, 0.60]: such a result is defined as maintaining a moderate consistency with the actual result;
4. Falling within the range of [0.61, 0.80]: such a result is defined as being highly consistent with the actual result;
5. Falling within the range of [0.81, 1]: such a result is defined as keeping almost complete with the actual result.

The classification training results of the four models are shown in Table 2.

It can be seen that the two optimized BP neural networks of GA-BP and PSO-BP have reduced simulation errors by 0.6351 and 0.7558 compared with the traditional BP neural network, respectively, and the Kappa coefficients of the two optimized BP neural networks of GA-BP and PSO-BP are the same. Both are 0.6, and the degree of agreement with the actual results is better than that of the traditional BP neural network, but the two optimized BP neural networks of GA-BP and PSO-BP are not much different from the perspective of simulation error or Kappa coefficient. It is impossible to distinguish which one is more suitable for classifying the images of the training area and the test area. For the GA-PSO-BP neural network remote sensing image classification method proposed in this article, the simulation error is significantly lower than those of the traditional BP, GA-BP, and PSO-BP neural networks. The Kappa coefficient of GA-PSO-BP neural network is 0.77.

### 4.5. Dynamic Analysis of Desertification Images

**Figure 10:**
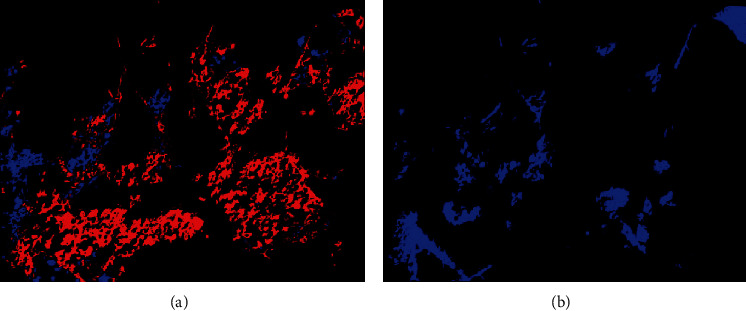
Changes in desertification land from 2010 to 2020. ^*∗*^Blue is decreasing area, red is increasing area, and black is nondesertification land. (a) 2010–2015. (b) 2015–2020.

This article selects the Horqin area in eastern Inner Mongolia to conduct desertification image dynamic analysis. The image change monitoring method adopted is the classification postprocessing method. The traditional image difference method and the implementation platform is ENVI5.3. The purpose of the difference method is to highlight the changed part of the image. To achieve the purpose of land change monitoring, Figure 10 shows the results of the change monitoring. The red part of the figure represents the increase of desertified land from 2010 to 2020, and the blue part of the figure represents the decrease of desertified land from 2010 to 2020. Statistics show that, from 2010 to 2020, the area of desertified land in the test area increased by 296130 pixels, totaling 1.593 km^2^, and decreased by 6,107 pixels, totaling 0.033 km^2^. The final increase in area is 1.56 km^2^.

Statistics show that, from 2010 to 2020, the area of desertified land in the test area decreased by 210,348 pixels, totaling 1.131 km^2^, and there was no increase in area. From 2010 to 2020, the area of desertified land in the test area showed a trend of first increasing and then decreasing.

## 5. Conclusion

For remote sensing images, this paper constructs a GA-PSO-BP analysis model based on BP neural network, genetic algorithm, and particle swarm optimization algorithm and compares the classification training accuracies of the four models of BP, GA-BP, PSO-BP, and GA-PSO-BP; GA-PSO-BP was selected for dynamic analysis of desertification images, and the results showed the following:

1. By comparing the regional classification training accuracies of the four models of BP, GA-BP, PSO-BP, and GA-PSO-BP, the GA-PSO-BP neural network remote sensing image classification method proposed in this paper is simple and easy to operate. Compared with traditional remote sensing image classification methods and traditional neural network classification methods, the classification accuracy of remote sensing effects is improved.
2. Carrying out desertification analysis on remote sensing images of Horqin area, from 2010 to 2015, the desertified land area in the test area increased by 1.56 km^2^; from 2015 to 2020, the desertified land area in the test area decreased by 1.131 km^2^, and the area of desertified land in the test area from 2010 to 2020 showed a trend of increasing first and then decreasing, which is consistent with the actual situation. The GA-PSO-BP remote sensing image classification model has a good performance portability.

## Figures and Tables

**Table 1:** Introduction to commonly used functions of BP neural network.

Function category	Function name	Features
Network creation function	Newcf	Create a multilayer feedforward network
	Newff	Create a feedforward network
Network initialization function	Initwb	Initialize the main structural parameters of the network
Transfer function	Logsig	S-type transfer function (logarithm)
	Tansig	S-type transfer function (tangent)
Learning function	Learngd	Gradient descent learning function
	Learngdm	Gradient descent (momentum learning) function
Training function	Traind	Training weight/threshold
	Adapt	Adaptive function
Simulation function	Sim	Neural network simulation
	Mse	Mean square error function

**Table 2:** Comparison of training results of each model.

Model	Kappa	OA	Misclassification error	Omission error	Simulation error
BP	0.47	0.56	0.007	0.01	12.3158
GA-BP	0.6	0.70	0.006	0.05	11.6807
PSO-BP	0.6	0.68	0.005	0.05	11.5600
GA-PSO-BP	0.77	0.82	0.004	0.06	10.0104

## Data Availability

The dataset can be accessed upon request.
